# Comparative efficacy and safety of different hemostatic methods in total hip arthroplasty: a network meta-analysis

**DOI:** 10.1186/s13018-018-1028-2

**Published:** 2019-01-04

**Authors:** Zhihu Zhao, Jianxiong Ma, Xinlong Ma

**Affiliations:** 1 Department of orthopedics, Tianjin Hospital, Tianjin, China; 2 Tianjin Institute of Orthopedics in Traditional Chinese and Western Medicine, Tianjin Hospital, NO.155 Munan Road, Tianjin, 300050 China

**Keywords:** Tranexamic acid, Fibrin sealant, Total hip arthroplasty, Blood loss

## Abstract

**Background:**

It is unclear which kind of interventional therapy is the best when reducing blood loss in patients prepared for total hip arthroplasty (THA). We performed this network meta-analysis to rank the best intervention arm for blood loss control in THA patients.

**Methods:**

We searched electronic databases about randomized controlled trials (RCTs) to compare three treatments (topical tranexamic acid (TXA), intravenous TXA, and topical fibrin sealant (FS)) versus placebo for the people prepared for THA. Traditional and network meta-analyses were performed. The quality assessment was conducted using Cochrane Collaboration’s tool. The network meta-analysis was conducted using Stata 13.0 software.

**Results:**

Finally, a total of 32 RCTs were included in this network meta-analysis. Topical TXA, intravenous TXA, and topical FS significantly decreased the need for transfusion and total blood loss when compared with placebo. And intravenous TXA ranks the first hemostasis agent for reducing the need for transfusion and total blood loss. There was no significant difference between these three treatments (intravenous TXA, topical TXA, and topical FS) in the occurrence of deep venous thrombosis (DVT).

**Conclusion:**

Intravenous TXA may be the best way to reduce the need for transfusion and total blood loss. More direct studies that focused on topical TXA versus FS are needed in the future.

## Introduction

Total hip arthroplasty (THA) is associated with considerable blood loss, which can lead to a need for transfusion. It is reported that perioperative blood loss in THA can be as much as 700–2000 ml, and subsequently, 16 to 37% of patients need blood transfusion [1, 2]. Blood transfusion has several serious complications, such as virus transmission and immunological reaction [3, 4]. What is more, the economic burden caused by blood transfusion will be increased correspondingly. Substantial blood loss was mainly caused by the osteotomy of the femoral and surgical trauma and fibrinolysis. In order to reduce blood loss, several strategies have been managed to inhibit the fibrinolysis and surgical bleeding. Fibrin sealant (FS) is composed of fibrinogen and thrombin that mainly derived from human blood products [5, 6]. When those components mixed, fibrin formed and crosslinked directly with tissue collagen [7]. Tranexamic acid (TXA) is a synthetic amino acid, and its structure is analogous to lysine that can competitively inhibit plasminogen and reduce fibrinolysis locally [8]. There are two main administration routes to the management of TXA: topical TXA and intravenous TXA [9]. Clinical studies and meta-analysis found that both the topical and intravenous TXA can reduce blood loss without sacrificing the safety [10]. And several studies have identified the efficacy and safety of FS for reducing perioperative blood loss in THA. In the current clinical practice, which hemostasis agents were the most effective was in debate. In addition, the meta-analysis comparing topical versus intravenous TXA in THA was limited. The purpose of this network meta-analysis was to compare the efficacy and safety of the three treatments (FS, topical TXA, and intravenous TXA) for patients prepared for THA. Our intention was to provide hierarchies of the need for transfusion, total blood loss, and incidence of deep venous thrombosis.

## Methods

### Criteria for considering studies

We only included RCTs which compared the need for transfusion, total blood loss, blood loss in drainage, and occurrence of DVT of the three main interventions (FS, topical TXA, and intravenous TXA) in people prepared for unilateral THA. Studies were included in the systematic review if they met the criteria: (1) primary unilateral THA; (2) RCTs; (3) intervention including FS, topical TXA, intravenous TXA, and control group; and (4) at least included one of the following outcomes: total blood loss, need for transfusion, and occurrence of deep venous thrombosis (DVT).

Trials were excluded if they (1) were meetings, letters, and protocols; (2) had repeated data or without insufficient data for meta-analysis; and (3) were retrospective design and prospective cohort studies.

### Search methods and study selection

We searched PubMed, Embase, Cochrane Central Register of Controlled Trials (CENTRAL), Google Scholar, and Web of Science from inception to August 2018. Keywords and MeSH terms including “total hip arthroplasty”; “total hip replacement”; “THA”; “THR”; “Arthroplasty, Replacement, Hip”[Mesh]; “fibrin glue”; “fibrin sealant”; “fibrin tissue adhesive”; “Fibrin Tissue Adhesive[Mesh]”; and “tranexamic acid” were used in the search strategy. We also viewed a systematic review and meta-analysis for any omissive papers. Two independent authors selected the included studies based on the title and abstract. Any disagreement about whether included or not was resolved by a discussion or consulted to a senior reviewer.

### Data collection and quality assessment

Two reviewers (Zhihu Zhao, Xinlong Ma) used a standardized form to extract data from the included studies. Information included study, sample size, comparators, study design, male patients, mean age, bone cement (cemented or uncemented), and dose of interventions. Meanwhile, we collected data about final outcomes: need for transfusion, total blood loss, and the occurrence of DVT. When relevant data was missing or needed to be identified, attempts were made to connect with the corresponding author by e-mail.

Cochrane risk of bias tool was used to assess the risk of bias. A total of seven domains were assessed and classified as low, unclear, and high risk of bias according to the suggestion of Cochrane risk of bias tool.

### Data analysis

Data were recorded into Microsoft® Excel (Microsoft Corporation, Redmond, WA, USA) by two reviewers (Zhihu Zhao and Xinlong Ma). If there are differences between reviewers, re-review the literature to resolve. For continuous data (total blood loss), the mean difference (MD) with 95% confidence interval (CI) was used for direct comparisons. For network meta-analysis, MD with 95% credible intervals (CrI) was calculated by Stata software. Dichotomous data (need for transfusion and the occurrence of DVT) were used for odds ratio (OR) with 95% CI or 95% CrI to express indirect comparisons. Anna Chaimani model for network meta-analysis was used as previously described [11, 12]. Briefly, we calculate the inconsistency factor (IF) and its 95% confidence interval (IF) to evaluate the consistency of each closed loop. When the lower limit of the 95% confidence interval is equal to 0, it was considered to be consistent. Otherwise, there is a significant inconsistency in the closed loop.

## Results

### Study identification and selection

**Fig. 1:**
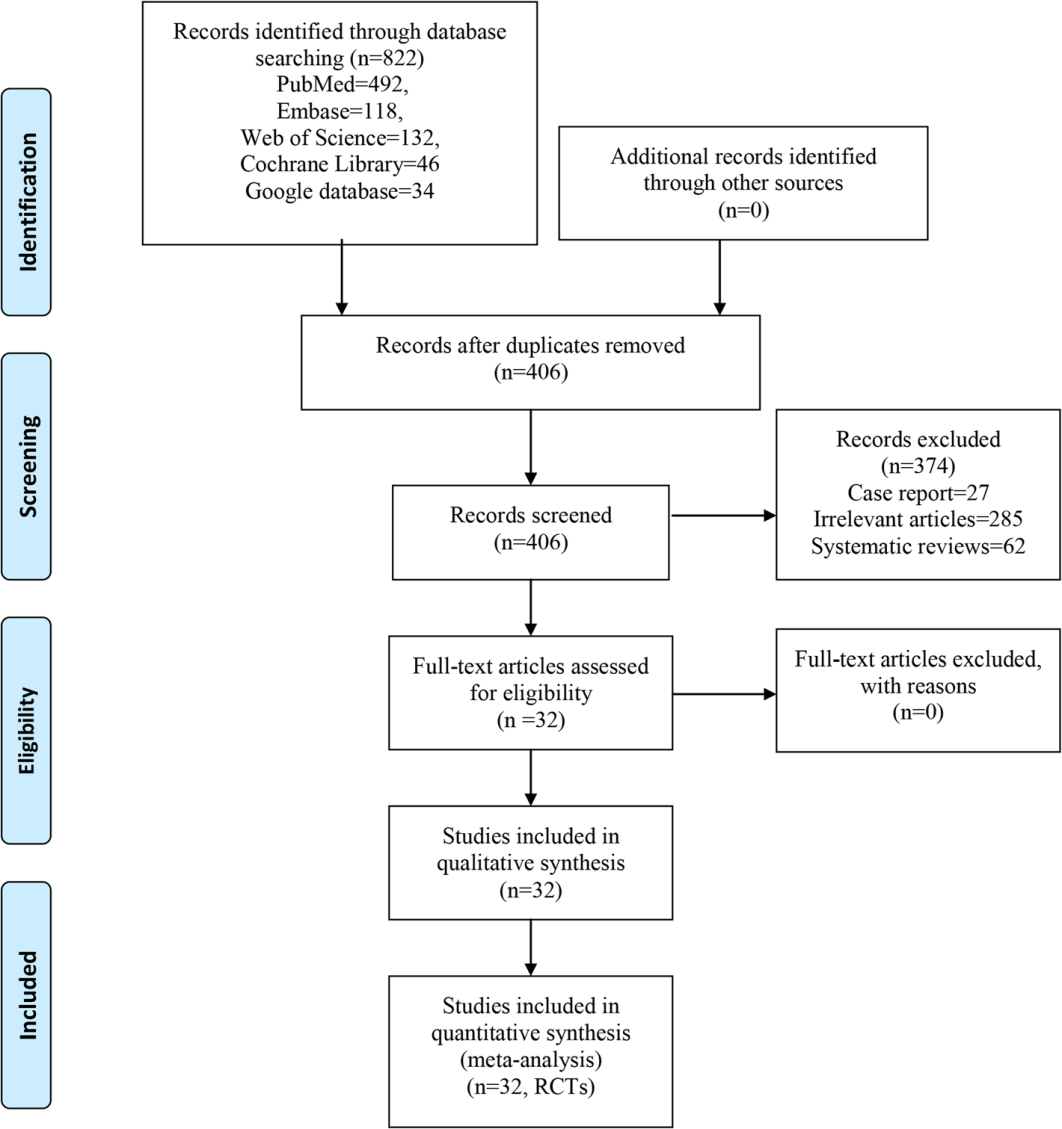
Flow diagram for the included studies

The literature search strategy process was shown in Fig. 1. Initially, we identified a total of 822 papers from electronic databases, and no additional records identified from other sources. After the duplicates were removed, a total of 406 papers were going to the next process. After scanning the titles and abstracts of these papers, 374 papers were excluded. In total, 32 studies were included in the meta-analysis [13–44].

### Study characteristics and risk of bias in included studies

**Fig. 2:**
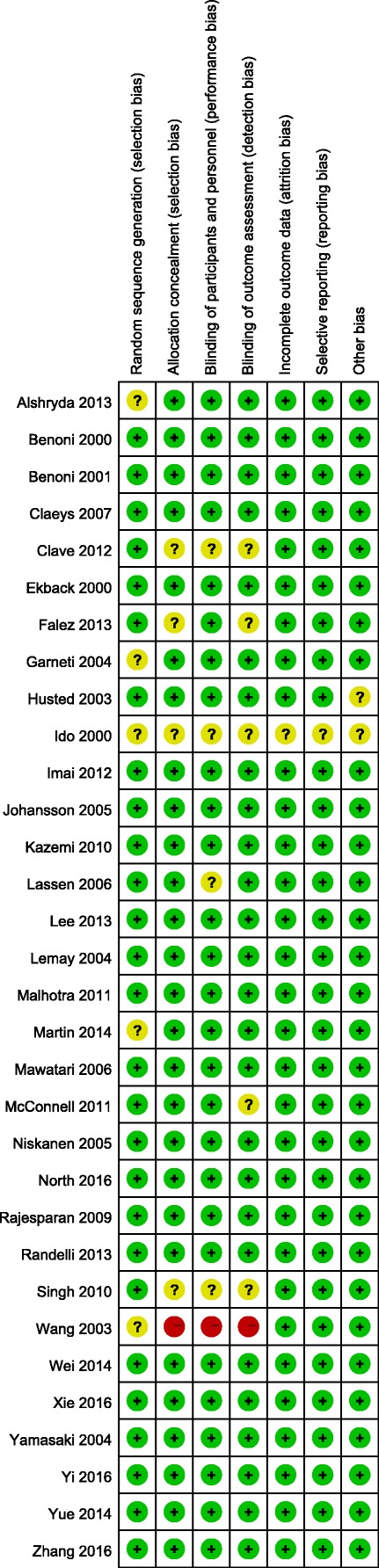
Risk of bias summary of the included studies. plus sign indicates low risk of bias; minus sign indicates high risk of bias; question mark indicates unclear risk of bias

**Fig. 3:**
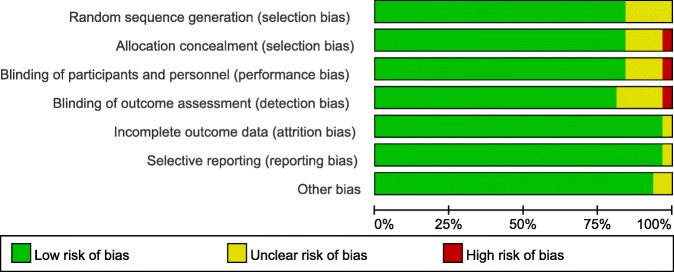
Risk of bias summary

As illustrated in Table 1, all the included studies provide detailed information about the general characteristics of the studies. A total of 31 clinical trials with 2476 patients were finally included in the meta-analysis. The sample size ranged from 20 to 102, and the age of the patients ranged from 45.4 to 73. Risk of bias summary and risk of bias graph can be seen in Figs. 2 and 3, respectively.

**Table 1:** The general characteristic of the included studies

Study	Sample size	Comparators	Study design	Male, %	Mean age	Bone cement	Dose
Benoni 2000	40 (20/20)	IV TXA/C	RCT	42.5	69.5/68	Cemented	10 mg/kg TXA
Ekbäck 2000	40 (20/20)	IV TXA/C	RCT	50	66.4/65.6	Cemented	10 mg/kg TXA
Ido 2000	40 (20/20)	IV TXA/C	RCT	NS	NS	Cemented	1000 mg TXA
Husted 2003	40 (20/20)	IV TXA/C	RCT	32.5	65/67	Cementless or hybird	10 mg/kg TXA
Lemay 2004	39 (20/19)	IV TXA/C	RCT	64.1	59.7/53.6	Cemented or cementless	10 mg/kg TXA
Garneti 2004	50 (25/25)	IV TXA/C	RCT	NS	67.6/69.6	Cemented	10 mg/kg TXA
Yamasaki 2004	40 (20/20)	IV TXA/C	RCT	67.5	55.5/61.2	Cementless	1 g TXA
Johansson 2005	100 (47/53)	IV TXA/C	RCT	43	69/68	Cemented	15 mg/kg TXA
Niskanen 2005	39 (19/20)	IV TXA/C	RCT	33.3	66/65	Cemented	10 mg/kg TXA
Claeys 2007	40 (20/20)	IV TXA/C	RCT	30	73/68	Hybrid	15 mg/kg TXA
Rajesparan 2009	73 (36/37)	IV TXA/C	RCT	35.6	67.5/67.7	Cemented, cementless or Hybrid	1 g TXA
Kazemi 2010	64 (32/32)	IV TXA/C	RCT	67.2	46.6/45.4	Cementless	15 mg/kg TXA
Singh 2010	42 (21/21)	IV TXA/C	RCT	45.2	69/73	Cemented or cementless	10 mg/kg TXA
McConnell 2011	66 (22/22/2)	IV TXA/FS/C	RCT	31.8	NS	Cemented	10 mg/kg TXA/10 ml FS
Malhotra 2011	50 (25/25)	IV TXA/C	RCT	44	52.6/54.7	Cementless	15 mg/kg TXA
Clave 2012	70 (37/33)	IV TXA/C	RCT	35.7	69/73	Cementless	1 g TXA
Norio 2012	117 (95/22)	IV TXA/C	RCT	17.9	64.4/60.2	Cementless	1 g TXA
Falez 2013	69 (31/38)	FS/C	RCT	NS	NS	Cementless	10 ml FS
Lassen 2006	69 (33/36)	FS/C	RCT	39.1	67.1/63.1	Cemented or cementless	NS
Mawatari 2006	100 (50/50)	FS/C	RCT	NS	60/60	Cementless	10 ml FS
Randelli F 2013	70 (35/35)	FS/C	RCT	41.2	63.1/64.2	Cementless	10 ml FS
Wang 2003	81 (38/43)	FS/C	RCT	54.2	66.9/67.8	NS	10 ml FS
Xie 2016	140 (70/70)	IV TXA/T TXA	RCT	45	59.5/62.2	Cementless	1.5g IV TXA/3g T TXA
Wei 2014	303 (100/102/101)	IV TXA/T TXA/C	RCT	37.3	63.6/60.2/63.9	Cementless	3g IV TXA/3g T TXA
North 2016	139 (70/69)	IV TXA/T TXA	RCT	55.5	64.1/65.7	Cementless	2g IV TXA/2 g T TXA
Zhang 2016	75 (25/25/25)	IV TXA/T TXA/C	RCT	52	44.5/44.3/43.4	Cementless	1g IV TXA/1 g T TXA
Martin 2013	50 (25/25)	T TXA/C	RCT	38.7	62.9/63.9	Cemented	2g T TXA
Alshryda 2013	161 (80/81)	T TXA/C	RCT	45.9	66/63	Cementless	NS
Yue 2014	101 (52/49)	T TXA/C	RCT	52.1	60.9/63.7	Cementless	3 g T TXA
Yi 2016	100 (50/50)	IV TXA/C	RCT	53	54/56.6	NS	15 mg/kg TXA
Lee 2013	68 (34/34)	IV TXA/C	RCT	NS	51.4/52.8	Cementless	15 mg/kg TXA
Benoni 2001	38 (18/20)	IV TXA/C	RCT	50	66/68	Cemented	10 mg/kg TXA

*IV* intravenous, *T* topical, *TXA* tranexamic acid, *C* control, *FS* fibrin sealant, *NS* not stated, *RCT* randomized controlled trials

### Effects of interventions on the need for transfusion

**Fig. 4:**
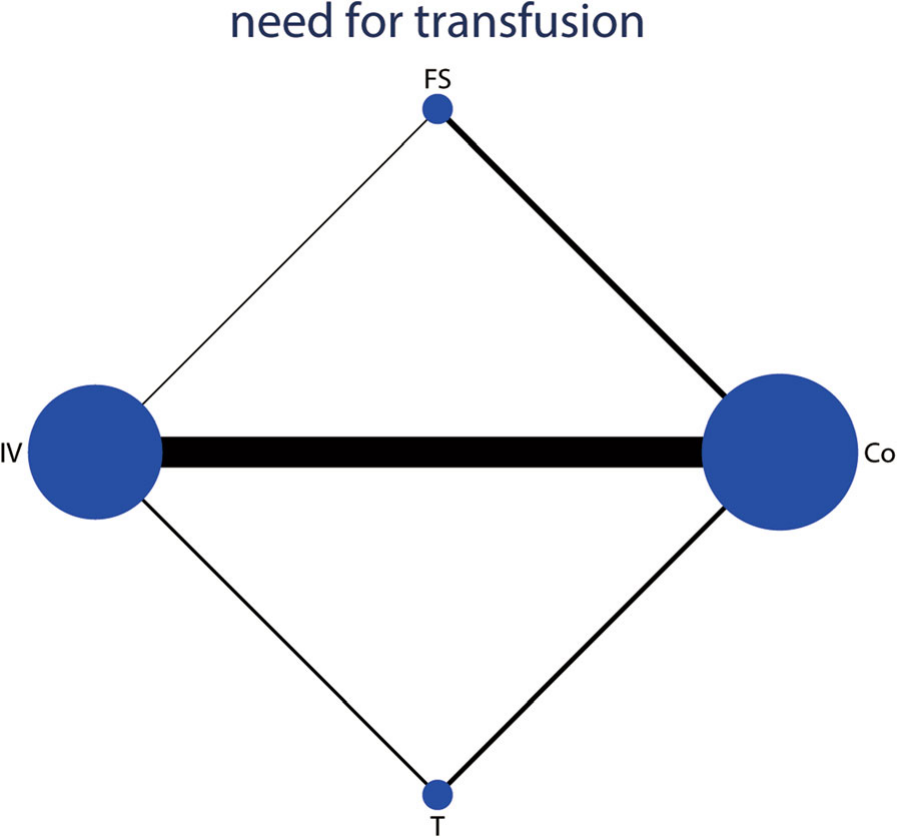
Network of treatment comparisons for the need for transfusion

**Fig. 5:**
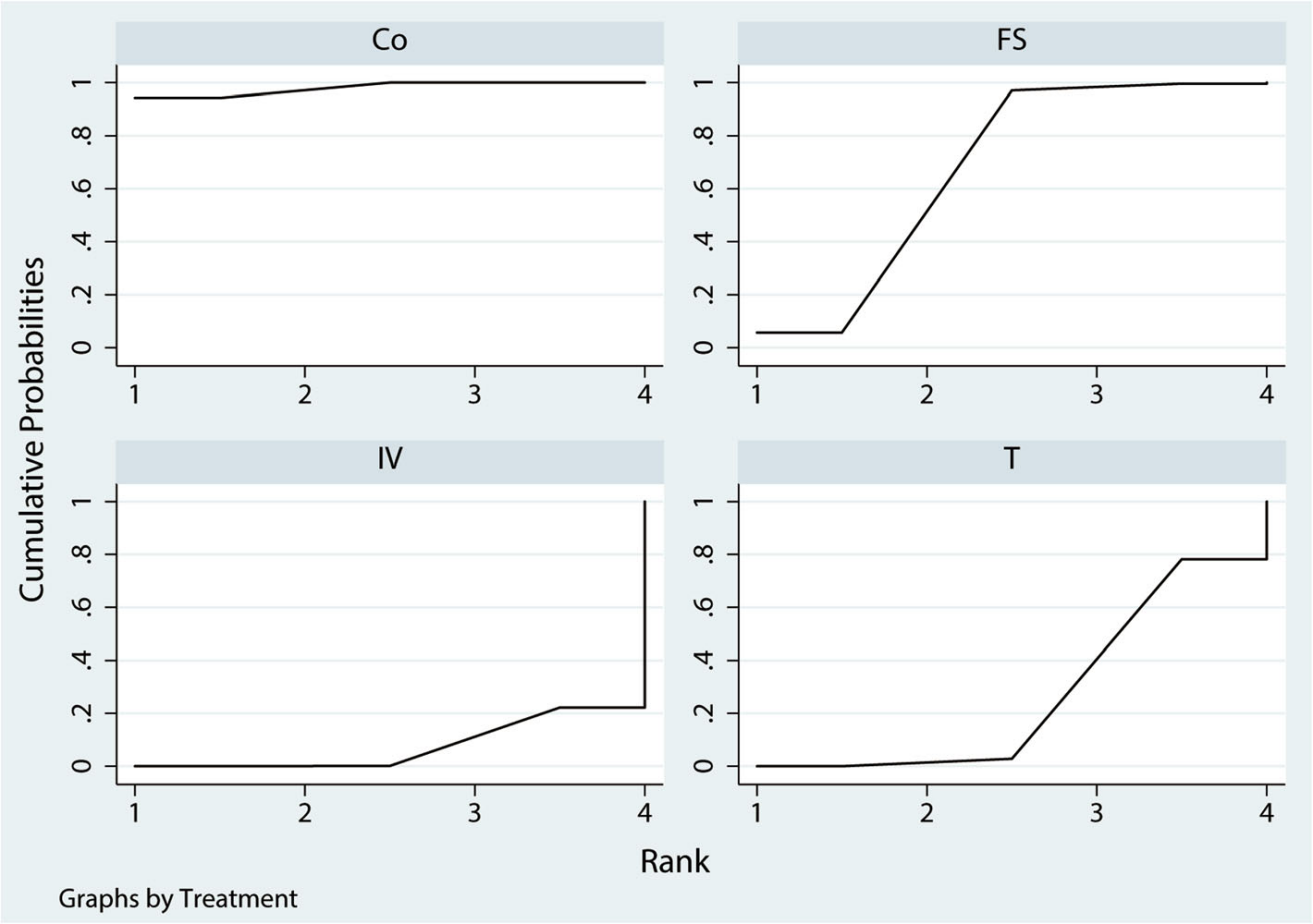
Ranking of treatment strategies based on the probability of their effects on the outcome of the need for transfusion

The network of four interventions on the need for transfusion was shown in Fig. 4. Table 2 provided hierarchies of effect size on the need for transfusion. Ranking graph of the distribution of probabilities on the need for transfusion was shown in Fig. 5. The direct and indirect comparisons indicated IV TXA, T TXA, and FS significantly decreased the need for transfusion compared with the control group. Based on SUCRA, control (0.97) ranked the first, the second was FS (0.66), the third was T TXA (0.23), and the last was IV TXA group (0.14).

**Table 2:**
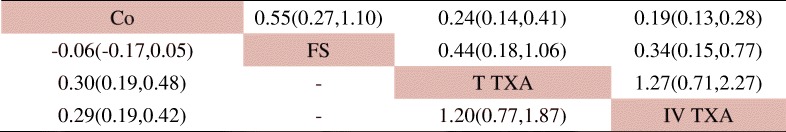
Need for transfusion of difference treatments

For need for transfusion, mean difference (MD) lower than 0 favor the column-defining treatment. Direct comparisons were shown in the upper right. Indirect comparisons were shown in the bottom left. The number which was painted by a style of overstriking indicated there was a significant difference between the two treatments. Co, control; FS, fibrin sealant; T TXA, topical tranexamic acid; IV TXA, intravenous tranexamic acid

### Effects of interventions on the total blood loss

**Fig. 6:**
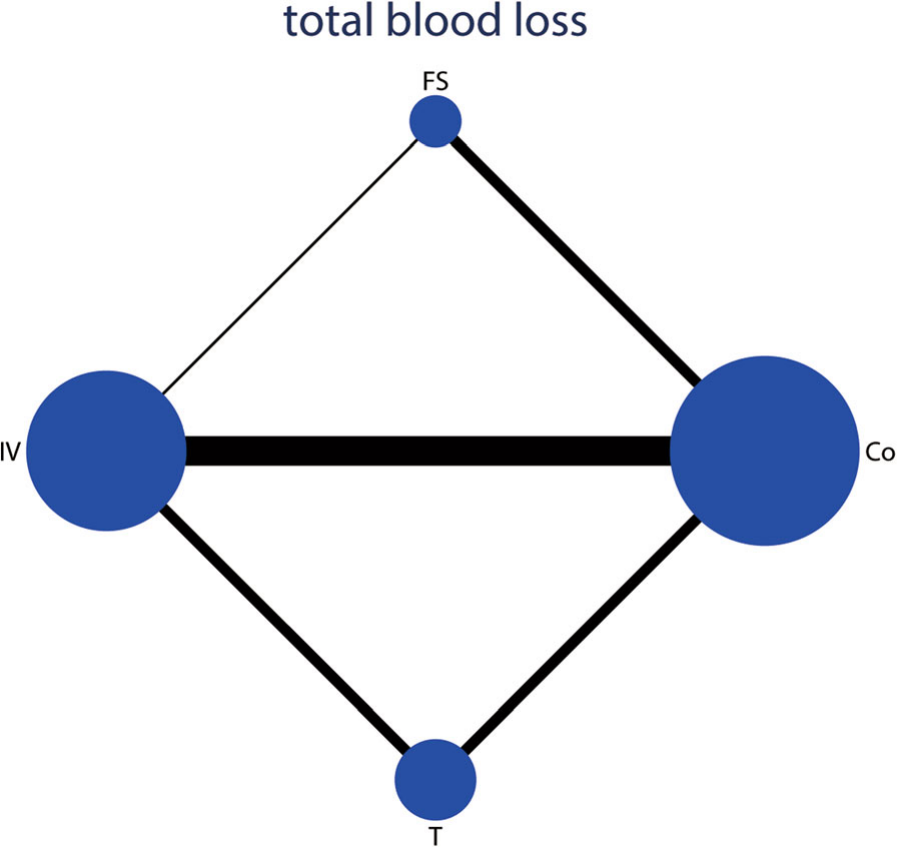
Network of treatment comparisons for total blood loss

**Fig. 7:**
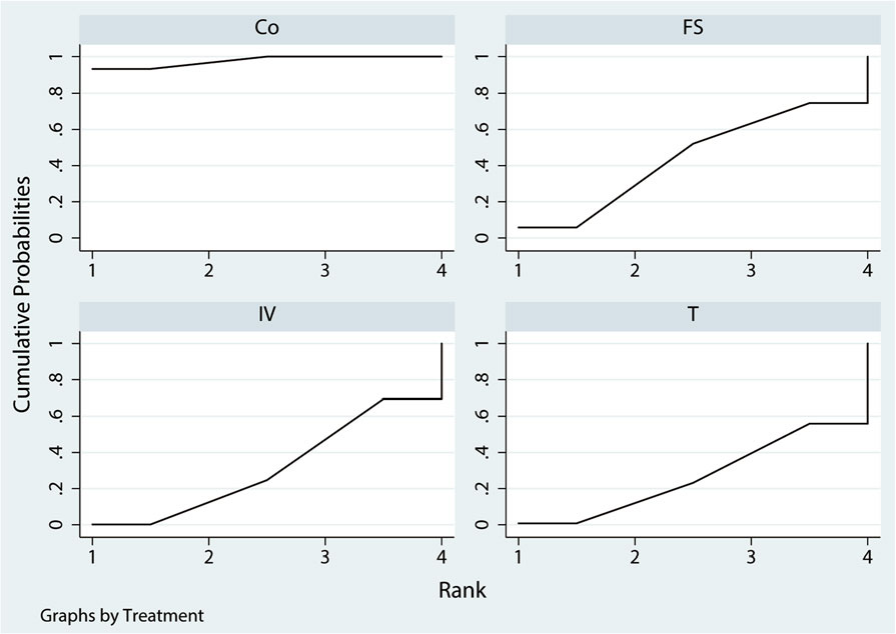
Ranking of treatment strategies based on the probability of their effects on the outcome of total blood loss

A total of 1287 THAs (IV TXA = 401, T TXA = 386, FS = 500, control = 562) were included for the analyses of total blood loss. The network of comparisons on total blood loss was shown in Fig. 6. Table 3 provided hierarchies of effect size on total blood loss. Figure 7 showed the ranking graph of the total blood loss between these treatments. The direct and indirect meta-analyses indicated IV TXA, T TXA, and FS significantly decreased total blood loss compared with the control group. Based on SUCRA value, Control (0.97) ranked the first, the second was FS (0.44), the third was T TXA (0.32), and the last was IV TXA group (0.27).

**Table 3:**
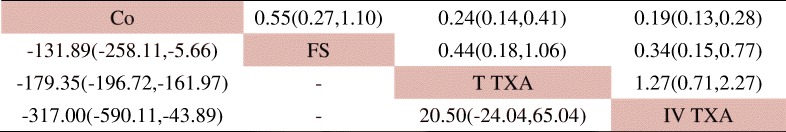
Total blood loss of difference treatments

For total blood loss, mean difference (MD) lower than 0 favor the column-defining treatment. Direct comparisons were shown in the upper right. Indirect comparisons were shown in the bottom left. The number which was painted by a style of overstriking indicated there was a significant difference between the two treatments. Co, control; FS, fibrin sealant; T TXA, topical tranexamic acid; IV TXA, intravenous tranexamic acid

### Effects of treatments on the DVT

**Fig. 8:**
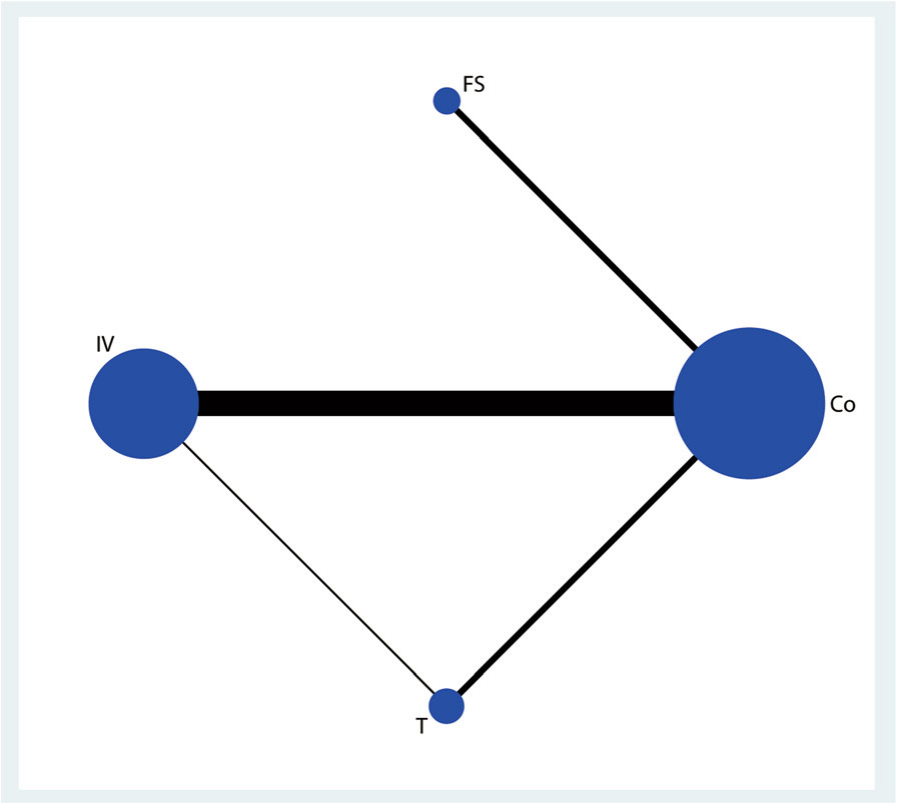
Network of treatment comparisons for the occurrence of DVT

**Fig. 9:**
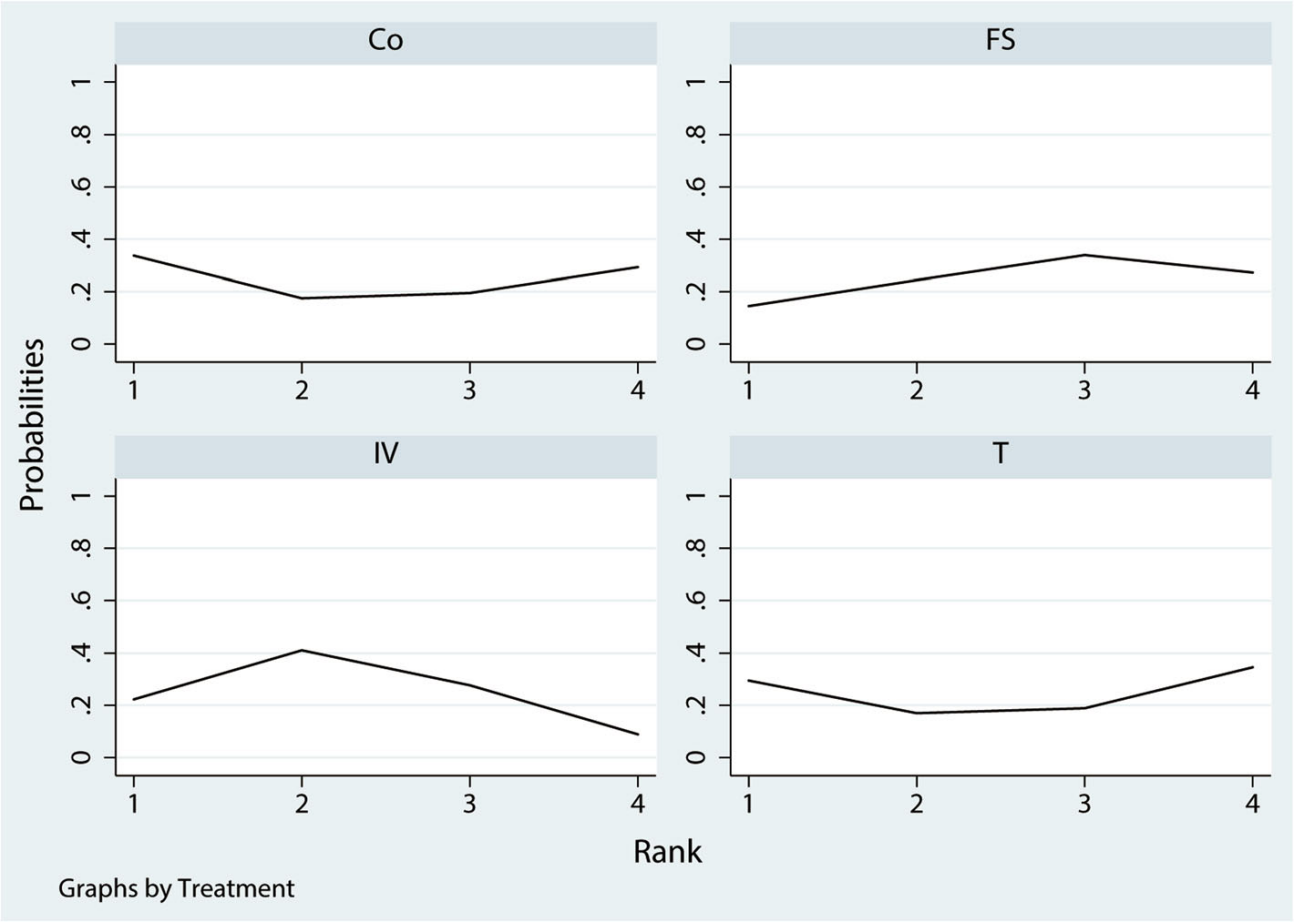
Ranking of treatment strategies based on the probability of their effects on the outcome of the occurrence of DVT

A total of 476 patients were assigned to IV therapy and 297 to topical therapy, 556 patients were assigned to the FS group, and 713 patients were assigned to control therapy. The network of 4 comparisons (IV TXA, T TXA, FS, and control) on the occurrence of DVT was shown in Fig. 8. We also made a ranking graph of the distribution of probabilities on the occurrence of DVT in Fig. 9. Based on SUCRA, Co (0.67) ranked the first, the second was T TXA (0.56), the third was FS (0.52), and the last was IV TXA group (0.48).

### Small-study effect and inconsistency test

**Fig. 10:**
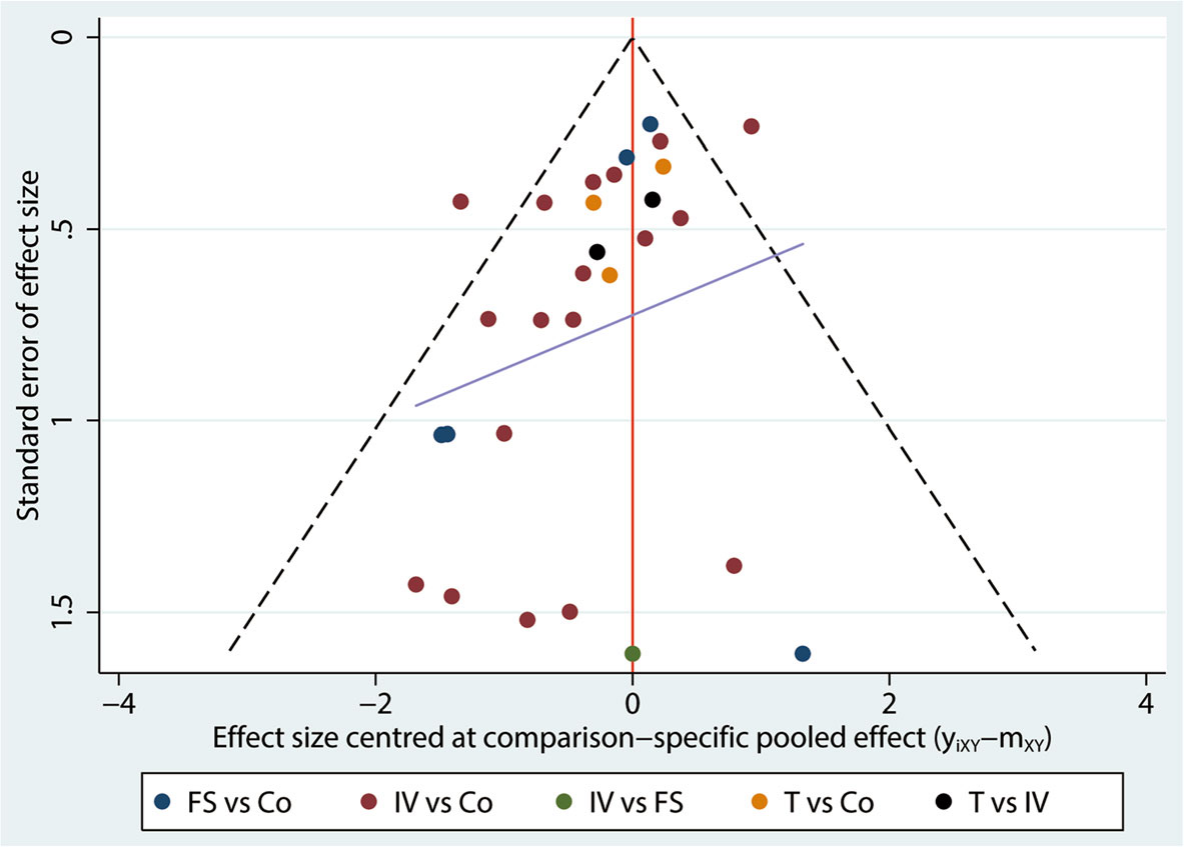
Funnel plot of the need for transfusion

Figure 10 shows that the funnel plot is symmetrical, indicating there is no publication bias in this network meta-analysis. Inconsistency test between direct and indirect comparisons revealed that the statistical inconsistency in the current meta-analysis was generally low because the CI values included zero.

## Discussion

This is the first systematic review and network meta-analysis that provided hierarchies for the need for transfusion, total blood loss, and the occurrence of DVT comparing two main hemostasis agents (FS and TXA) after THA. All the included studies were RCTs, and the general characteristic was comparable that all patients were old patients prepared for unilateral THA. There were several strengths in this network meta-analysis: (1) comprehensive search strategy by two authors was used to increase the robustness of the search results; (2) traditional and network meta-analysis were both performed to exhibit the evidence for hemostasis in THA patients; (3) we used SUCRA to rank these interventions; and (4) only RCTs were included in this article.

The meta-analysis indicated that (1) FS, IV TXA, and topical TXA can reduce total blood loss and need for transfusion after THA; (2) for decreasing the need for transfusion, the ranking of treatments was IV TXA, topical TXA, FS, and control; (3) for reducing total blood loss, the ranking of treatments was topical TXA, IV TXA, FS, and control group; (4) direct comparison indicated that there is no significant difference between IV TXA and topical TXA; (5) direct comparison showed that FS, IV TXA, and topical TXA can decrease blood loss and the need for transfusion compared with the control group; and (6) there is no direct comparison between topical TXA or intravenous TXA and FS.

The results of current network meta-analysis indicated that IV or topical TXA is the most preferable hemostasis agent in THA. The efficacy of hemostasis was tested by the need for transfusion and total blood loss. Though the blood transfusion trigger is different between the included studies, the consistency test was performed and the included studies are consistent. There is a contradictory result for IV TXA versus topical administration TXA. As for the need for transfusion, IV TXA ranks the first, and for total blood loss, topical TXA ranks the first. Based on these results, a direct comparison was conducted between topical and intravenous TXA for THA. The results indicated that there is no significant difference between topical TXA and intravenous TXA in THA. These results were consistent with the previous meta-analysis. Until now, there is no evidence indicating that IV TXA is superior to topical TXA. Only one trial directly compared IV TXA with FS since there is no relevant data for blood loss and the need for transfusion for meta-analysis. Indirect data showed that whether IV TXA or topical TXA shows better hemostasis effects than FS. For TXA, there are no actual protocols that what dose is effective and safe. In the previous studies, intravenous 10 mg/kg, 15 mg/kg, or multiple doses are all been identified as effective and safe. The dose of topical TXA ranged from 1 to 3 g, and the administration routes included intra-articular and drain tube.

There is a previous meta-analysis comparing TXA with FS in total knee arthroplasty and found that there is no significant difference between the two agents. The meta-analysis including limited studies and non-RCTs will make the large heterogeneity for the final results. Another factor that affects the alternative choice for hemostasis agent is the price. FS is considerably costlier than TXA. The therapeutic dose of TXA (10 mg/kg) will cost about 8€, while FS will cost between 450€ and 675.00€. FS was manufactured from human plasma products, and in common with other blood-derived products, there is a risk of transmission of disease but concern may remain relating to unknown vectors.

There were several limitations for this meta-analysis: (1) the indirect comparison between FS and IV TXA was limited in total blood loss and the occurrence of DVT and thus may affect the precision of the final outcomes; (2) the follow-up in these studies was relatively short, and long-term follow-up was needed to identify the potential omitted complications; and (3) allocation concealment in some studies were limited and may cause heterogeneity between the studies.

In summary, our finding indicated that IV TXA was the most preferable hemostasis method for blood loss control in THA patients. And the use of IV TXA will not increase the occurrence of DVT. More direct evidence was needed to identify the optimal method for blood loss control in THA patients.

## Abbreviations

CENTRAL: Cochrane Central Register of Controlled Trials
CI: Confidence interval
CrI: Credible intervals
DVT: Deep venous thrombosis
FS: Fibrin sealant
MDs: Mean differences
RCTs: Randomized controlled trials
THA: Total hip arthroplasty
TXA: Tranexamic acid
